# Early- and Long-Term Outcomes of Mitral Valve Repair in a Low-Volume Centre in the Caribbean

**DOI:** 10.21470/1678-9741-2020-0421

**Published:** 2022

**Authors:** Richard A. E. Ramsingh, Gianni D. Angelini, Risshi D. Rampersad, Natasha C. Rahaman, Giovanni Teodori

**Affiliations:** 1 Cardiology Department, Caribbean Heart Care Medcorp, St. Clair Medical Centre, St. Clair, Port of Spain, Trinidad & Tobago.; 2 Cardiac Surgery Department, Caribbean Heart Care Medcorp, St. Clair Medical Centre, St. Clair, Port of Spain, Trinidad & Tobago.; 3 Bristol Heart Institute, Bristol University, United Kingdom.

**Keywords:** Mitral Valve Insufficiency, Mitral Valve Annuloplasty, Data Management, Reoperation, Echocardiography, Caribbean Region

## Abstract

**Introduction:**

This study examines early- and long-term outcomes of mitral valve repairs in a low-volume cardiac surgery centre in the Caribbean.

**Methods:**

Ninety-six consecutive patients underwent mitral valve repair from April 2009 to December 2018. Patients were divided into two groups: functional mitral regurgitation requiring simple mitral annuloplasty (FMR, n=63) or structural degenerative mitral regurgitation requiring more complex repair (DMR, n=33). Data collected prospectively were retrospectively analysed from the unit-maintained cardiac surgery database.

**Results:**

Thirty-day mortality in the whole series was 2.1%, with 3% in the FMR group and 0% in the DMR group. Early post-operative echocardiography in the FMR group demonstrated 51 patients (83.6%) without mitral regurgitation, 8 patients (13.1%) with trivial to mild regurgitation, and 2 patients (3.3%) with moderate regurgitation. However, at a mean follow-up of 98.2±50.8, only 21 patients (42.8%) were in NYHA class I, with 7 (14.2%) in class II, 16 (32.6%) in class III, and 5 (10.2%) in class IV. There were 9 cardiac-related deaths at final follow-up, with freedom from re-operation and survival of 98% and 75.6%, respectively. In the DMR group, early post-operative echocardiography demonstrated 29 patients (87.9%) without mitral regurgitation, 3 patients (9.1%) with trivial regurgitation and 1 patient (3.0%) with mild regurgitation. At a mean follow-up of 114.1±25.4 months, there was a good functional post-operative status in this group with 93.3% in NYHA class I, and 6.7% in class II. No patient required reintervention, 96.3% of patients had mild or no mitral regurgitation and survival was 90.9%.

**Conclusion:**

Despite challenges of maintaining skills in a low-volume centre, mitral valve repair can be performed safely with good early- and long-term results.

**Table:** 

Abbreviations, acronyms & symbols	
CABG	= Coronary artery bypass grafting
DMR	= Degenerative mitral regurgitation
FMR	= Functional mitral regurgitation
LVEF	= Left ventricular ejection fraction
MIDA	= Mitral Regurgitation International Database
NYHA	= New York Heart Association

## INTRODUCTION

Mitral valve repair is the gold standard for the treatment of isolated severe mitral regurgitation secondary to degenerative mitral valve disease ^[1]^. Its superiority, by comparison with mitral valve replacement, has been demonstrated by many groups and is currently recommended by both US and European guidelines ^[1,2]^. However, the evolution of mitral valve repair has seen challenges and mastering the techniques requires a significant learning curve.

In recent years, US and European guidelines have described centres of excellence in mitral valve surgery as centres with high repair rates, low operative mortality, and a record of durable results. However, there is a paucity of literature on operative outcomes in low-volume centres. This warrants attention in the Caribbean as there are no high-volume mitral valve centres. 

The goal of our study, therefore, was to evaluate early- and long-term mitral valve repair results in functional and degenerative groups over a 10-year period at a low-volume centre in Trinidad and Tobago. 

## METHODS

### Patients and Study Protocol

From April 2009 to December 2018, 110 patients were scheduled for mitral valve repair at Caribbean Heart Care Medcorp Ltd, St. Clair, Trinidad and Tobago. After receiving approval from the institutional ethics committee, we reviewed prospectively entered data from the unit maintained cardiac surgery database of consecutive patients. In addition, all operative notes and discharge summaries were reviewed to cross-reference the database, input data that were missing from the database, and to collect supplementary surgical procedural data. Of the 110 patients in whom mitral valve repair had been planned, the repair was successful in 96 patients (87%). In 14 patients, mitral valve replacement was carried out, and these patients were not included in the study protocol. 

### Operative Techniques and Follow-Up

Decisions to repair were made intra-operatively following transoesophageal echocardiogram and exploration and inspection of the mitral valve. Three patients elected for right antero-lateral thoracotomy and the remaining 93 patients were operated through a median sternotomy.

Operations were performed under cardio-pulmonary bypass at 34 ºC and cold blood cardioplegic arrest. In the DMR group, correction of prolapsed leaflets was initially accomplished by using techniques described by Carpentier (mainly leaflet resection with sliding or folding annuloplasty), and, more recently, by chordal plication and supporting annuloplasty ring. Concomitant cardiac procedures were also performed as required (Table 1). Repair in the FMR group was performed using an annuloplasty ring only (Table 2). Most commonly used, the open Braile rigid ring (Braile Biomédica, São Paulo, Brazil) was modified by re-shaping it to adapt to the mitral valve annulus anatomy. Occasional annuloplasty ring options included the St. Jude Medical mitral annuloplasty ring (Minnesota, USA) and Dacron rings made from Dacron tube grafts (Terumo Corporation, Tokyo, Japan) ^[3]^. All patients received aspirin postoperatively. Intraoperative transoesophageal echo was performed before and after the procedure in all patients.

**Table 1:** Patients' characteristics.

	FMR (n=63)	DMR (n=33)	Whole series (n=96)
Age (years)	58.7±7.5	53.6±14.7	56.5±11.4
Male sex	44 (69.8)	22 (66.7)	66 (68.8)
Diabetes	35 (55.6)	8 (25.0)	43 (44.8)
Hypertension	40 (63.5)	10 (31.3)	50 (52.1)
Atrial fibrillation	0 (0)	7 (21.2)	7 (7.3)
Preoperative LVEF, %	39.4±12.1	61.9±9.7	47.4±15.6
**Non-isolated MV repair**			
CABG	47 (74.6)	7 (21.2)	54 (56.3)
AVR	11 (17.5)	0 (0)	11 (11.5)
Tricuspid annuloplasty	5 (7.9)	4 (12.1)	9 (9.4)
PFO closure	0 (0)	3 (9.1)	3 (3.1)
ASD repair	0 (0)	1 (3.0)	1 (1.0)
LAA ligation	0 (0)	1 (3.0)	1 (1.0)

Values are mean±SD, n (%). ASD=atrial septal defect; AVR=aortic valve replacement; CABG=coronary artery bypass grafting; DMR=degenerative mitral regurgitation; FMR=functional mitral regurgitation; LAA=left atrial appendage; LVEF=left ventricular ejection fraction; PFO=patent foramen ovale

**Table 2:** Surgical details.

Surgical approach	
Median sternotomy	93 (96.9)
Right thoracotomy	3 (3.1)
**DMR group (complex repair)**	
Alfieri edge-to-edge	1 (3.0)
Quadrangular resection of posterior leaflet	20 (60.6)
Resection of prolapsed part of leaflet	4 (12.1)
Suture of cleft of anterior leaflet	1 (3.0)
Repair of perforated anterior leaflet	1 (3.0)
Repair of perforated posterior leaflet	1 (3.0)
Repair of posterior leaflet without quadrangular resection	4 (12.1)
Repair of anterior leaflet without quadrangular resection	1 (3.0)
**FMR group (annuloplasty only)**	
Braile	58 (92.1)
St. Jude Medical	2 (3.2)
Dacron	3 (4.8)

Values are n (%). DMR=degenerative mitral regurgitation; FMR=functional mitral regurgitation

Echocardiographic follow-up was obtained only for the complex repair DMR group. Severity of regurgitation was assessed semi-quantitatively on a scale from 0 to 4 by Doppler echocardiography where 0: none; 1: trivial-to-mild, 2: moderate; 3: moderate-to-severe; and 4: severe. Grading of mitral regurgitation was based on jet area, vena contracta width, left atrial size, pulmonary venous flow, mitral valve morphology, regurgitant volume and effective regurgitant orifice area ^[4,5]^. Follow-up vital status was obtained from phone interviews, hospital clinical data and death certificates were consulted for attribution of cause of death. These included: post-operative re-operation, atrial fibrillation, myocardial infarction, thromboembolism, re-hospitalization, and pacemaker insertion.

### Statistical Analysis

Data were expressed as mean±SD, and categorical variables were reported as numbers and percentages. The main end points of interest were 30-day mortality, recurrence of moderate/severe mitral regurgitation, reoperation rates, and overall survival. Survival and freedom from recurrent moderate/severe regurgitation were assessed by the Kaplan-Meier method. Patients that did not reach the end point were censored at the end of study period. 

## RESULTS

### Functional Mitral Regurgitation (FMR)

Forty-four patients (69.8%) were male, with an average age of 58.7±7.5 years. Thirty-five (55.6%) had diabetes and 40 (63.5%) had hypertension. Mean preoperative left ventricular ejection fraction (LVEF) was 39.4% (range 20%-65%). All 63 patients with FMR underwent simple mitral valve annuloplasty using a modified Braile rigid ring (92.1%) plus concomitant procedure as required (*i.e*., CABG, tricuspid valve repair) (Table 1). There were two in-hospital deaths. 

**Fig. 1:**
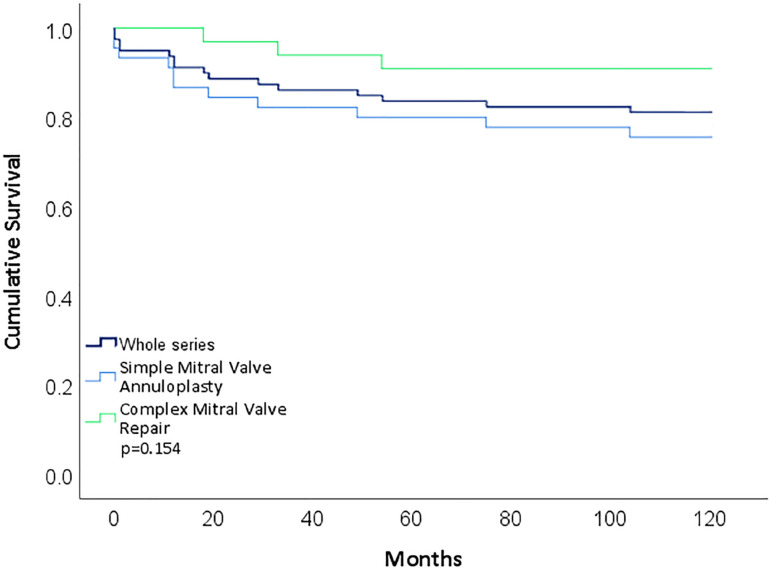
Cumulative survival probabilities. Kaplan-Meier estimate of survival function comparing whole series, FMR (simple), and DMR (complex) groups.

Early post-operative echocardiography demonstrated: 51 patients (83.6%) had no mitral regurgitation, 8 (13.1%) had trivial to mild regurgitation, and 2 (3.3%) had moderate regurgitation. Follow-up at a mean of 98.2±50.8 months was 92% complete (5 patients were lost to follow-up). Patients in this group, as expected because of the pathology, showed moderate improvement: 21 patients (42.8%) were in NYHA class I, with 7 (14.2%) in class II, 16 (32.6%) in class III, and 5 (10.2%) in class IV. There were 9 cardiac-related deaths in the last follow-up (Figure 1). One patient required a mitral valve replacement after 1 year (1.6%). Freedom from re-operation in the surviving patients was 98%. 

### Degenerative Mitral Regurgitation (DMR)

Twenty-two patients (66.7%) were male, with an average age of 53.6±14.7 years. Eight patients (24.2%) had diabetes and 10 (31.3%) had hypertension. Mean preoperative left ventricular ejection fraction (LVEF) was 61.9% (Table 1). Quadrangular resection of posterior leaflet with placement of an annuloplasty ring (Braile) was used in most patients, 20 (60.6%), 4 patients (12.1%) had posterior leaflet repair without resection, and 3 patients (9.1%) had anterior leaflet pathology (Table 2).

Early post-operative echocardiography demonstrated: 29 patients (87.9%) had no mitral regurgitation, 3 (9.1%) had trivial regurgitation and 1 (3.0%) had mild regurgitation. 

There were 3 cardiac-related deaths in the last follow-up (114.1±25.4 months, Figure 1). Of the 33 repairs, 28 patients (93.3%) were in NYHA class I, and 2 (6.7%) in NYHA class II. Ten patients (37%) had no mitral regurgitation, 16 had mild (59.3%) regurgitation, and 1 had moderate (3.7%) regurgitation. Three patients were either unable to or refused follow-up echocardiography (9.1%). No patient in the series required reintervention. 

Cumulative survival probability at 10 years was 81% in the whole series with 90.9% in the DMR group and 75.6% in the FMR group (Figure 1).

## DISCUSSION

Mitral valve repair is widely accepted by both European and US guidelines as the preferred treatment for primary mitral regurgitation ^[1,2]^. The MIDA (Mitral Regurgitation International Database) investigators in 2008 concluded that, among patients with degenerative mitral regurgitation with a flail leaflet, valve repair was associated with lower operative mortality, better long-term survival and fewer valve-related complications compared with mitral valve replacement ^[6]^. Since then, the concept of centres of excellence have evolved to set the standards for mitral valve repair. These criteria include high mitral valve repair volume, appropriate peri-procedural imaging capabilities and willingness to provide data regarding expected outcomes based on the centre’s recent experience, including repair, mortality and stroke rates, and repair durability ^[7,8]^. 

In our series, the results of mitral valve repair in patients who underwent either simple annuloplasty for functional mitral incompetence or complex repair in degenerative mitral valve incompetence were similar to those reported for high-volume centres of excellence. We observed no operative mortality, defined at 30 days following complex repairs in keeping with the accepted norm of repair ^[9-11]^. Simple repairs achieved a 3% operative mortality, also in line with the norm of those with ischemic functional mitral regurgitation ^[12,13]^. At follow-up of the degenerative complex repair functional status, freedom from reoperation and survival were also in line with previous reports of large, multi-centre registries ^[9,12,14,15]^. 

Maintaining the required skills for mitral valve repair is considered difficult for a surgeon who performs on average fewer than 10 procedures a year ^[16-18]^. This makes it rather challenging in the developing world where low-volume surgery is often the norm. 

Guidelines have moved toward earlier intervention for patients with asymptomatic severe mitral regurgitation only if surgery can be safely performed by an experienced team at a reference centre with a high likelihood of a durable repair. Achieving a durable, long-lasting mitral valve repair is therefore essential. In our series, we were able to achieve repair results like those reported by large institutions, despite being a ‘low-volume’ centre-defined as less than 25 mitral valve repairs per year. It is important to emphasise that these results were obtained by a single surgeon (GT) operating in a single experienced hospital. We further attribute these results to the establishment of a cardiac surgery program ^[19]^, with a dedicated multi-disciplinary team, including cardiology and critical care personnel optimally equipped to evaluate and manage these patients. 

In the Caribbean, trends of mitral valve repair remain unknown. In the wider world, many have accepted procedural volume as a proxy for quality ^[20]^. In a large, multicentre analysis by Chikwe et al. ^[21]^, it was found that individual surgeons’ mitral valve case volume has a significant impact on early- and long- term patients’ outcomes after mitral valve surgery. However, it is scarcely reported in the literature if centres with low-volume and a skilled surgeon could consistently reproduce these results. Furthermore, it is important to keep in mind that having surgery performed at a hospital conveniently located within the region is only beneficial if the outcomes are not inferior. Our data reflects the experience at a single institution and thus may not necessarily be generalisable. Nevertheless, our data demonstrates feasibility of performing complex mitral valve operations in the Caribbean. We believe that this will give reassurance to patients to undergo the procedure in their home country rather than travel to a foreign centre for expensive treatment.

## CONCLUSION

In conclusion, despite challenges in maintaining skills in a low-volume centre, valve repair for functional or degenerative mitral valve disease can be performed safely, with good early and long-term results.

**Table:** 

Authors' roles & responsibilities	
RAER	substantial contributions to the conception or design of the work; or the acquisition, analysis, or interpretation of data for the work; drafting the work or revising it critically for important intellectual content; final approval of the version to be published
GDA	substantial contributions to the conception or design of the work; or the acquisition, analysis, or interpretation of data for the work; drafting the work or revising it critically for important intellectual content; final approval of the version to be published
RDR	substantial contributions to the conception or design of the work; or the acquisition, analysis, or interpretation of data for the work; drafting the work or revising it critically for important intellectual content; final approval of the version to be published
NCR	substantial contributions to the conception or design of the work; or the acquisition, analysis, or interpretation of data for the work; drafting the work or revising it critically for important intellectual content; final approval of the version to be published
GT	substantial contributions to the conception or design of the work; or the acquisition, analysis, or interpretation of data for the work; drafting the work or revising it critically for important intellectual content; final approval of the version to be published
